# Exploring landscape of measles vaccination coverage: A step towards measles elimination goal in India

**DOI:** 10.1016/j.vaccine.2024.04.075

**Published:** 2024-06-20

**Authors:** Pritu Dhalaria, Pawan Kumar, Ajay Verma, Pretty Priyadarshini, Ajeet Kumar Singh, Bhupendra Tripathi, Gunjan Taneja

**Affiliations:** a Immunization Technical Support Unit, Ministry of Health & Family Welfare, Government of India, New Delhi 110070, India; b Immunization Division, Ministry of Health & Family Welfare, New Delhi 110011, India; c Department of Economics, Banaras Hindu University, Varanasi, Uttar Pradesh 221005, India; d Bill & Melinda Gates Foundation, New Delhi 110067, India

**Keywords:** Zero-dose, Measles, Vaccine, Elimination, Coverage

## Abstract

**Introduction:**

Measles remains a critical public health concern causing significant morbidity and mortality globally. Despite the success of measles vaccination programs, challenges persist, particularly in India. This study investigates dose-wise measles vaccination coverage and explores gaps in immunization focusing on zero-dose, one-dose, and two-dose coverage among children aged 24–35 months.

**Data sources and methodology:**

The National Family Health Survey 2019–21 (NFHS-5) served as the data source and the study analyzed information from 43,864 children aged 24–35 months. Sociodemographic variables such as birth order, wealth quintile, gender, social group, religion, residence, mother education, delivery-related factors, and media exposure were considered. Statistical analysis involved weighted estimates, chi-square tests, and multivariate multinomial logistic regression.

**Results:**

The study revealed that challenges persist in achieving optimal measles vaccination coverage. Analysis by sociodemographic factors highlighted disparities in coverage, with variations in zero dose prevalence across states and districts. The percentage of zero-dose children was significantly higher, with 11.5% of children in India remaining to receive any measles vaccination. Factors influencing vaccine coverage include birth order, age, wealth quintile, social group, religion, residence, maternal education, place of delivery, media exposure, and mode of delivery. The findings from the spatial analysis show the clustering of zero-dose children is high in the northeastern states of India.

**Discussion:**

Measles zero-dose children pose a significant obstacle to achieving elimination goals. Spatial analysis identifies clusters of unvaccinated populations guiding targeted interventions. The study aligns with global initiatives such as the Immunization Agenda 2030 emphasizing equitable vaccine access and discusses how India can tailor its strategies to achieve the goal. Lessons from polio eradication efforts inform strategies for measles elimination, stressing the importance of high-quality data and surveillance. The study underscores the urgency of addressing last-mile measles vaccination gaps in India. Spatially targeted interventions informed by sociodemographic factors can enhance immunization coverage. Achieving measles elimination requires sustained efforts and leveraging lessons from successful vaccination campaigns. The study findings have the potential to contribute to informed decision-making, supporting India's roadmap for the measles and rubella elimination goal.

## 1 Introduction

Measles is one of the contagious and life-threatening diseases that can be prevented by vaccines [1]. As per recent estimates from the World Health Organization (WHO), approximately 128,000 people died because of measles worldwide in 2021, largely constituting children who were either unvaccinated or partially vaccinated. Measles vaccination saved 56 million lives globally between 2001–2021. However, only 81 % of children received the first dose of measles vaccine by 12 months of age in 2021, which was the lowest since 2008 [2]. It is a significant cause of childhood morbidity and mortality in developing countries [3], [4].

The measles virus belongs to the family *Paramyxoviridae* which can remain viable in the air or any surface for hours [5]. Humans are the sole reservoir and the symptoms begin to appear after an average incubation period of ten days in the form of a high-grade exanthematous fever, cough, coryza, conjunctivitis, and maculopapular rash [6], [7]. After contracting measles, individuals often experience a period of immunosuppression, which leaves them vulnerable to opportunistic infections to other diseases in the long term. [8]. The case fatality ratio (CFR) related to measles has declined over the period but there are still large heterogeneities across the world starting from 0.79 % in developed countries to 7.67 % in developing countries [9].

After establishing the Expanded Program on Immunization (EPI) in 1974, WHO targeted measles as one of the first diseases under EPI. Between 2000–2020, around 31.7 million deaths due to measles have been averted globally as nations have concerted their efforts on governance, stewardship, human resources, and financing toward measles vaccination programs and their integration into their national immunization program [10], [11], [12].

Innovations in the immunization field led to a reduction in the prevalence of vaccine-preventable diseases. In 2021, globally, the annual reported incidence of measles is 16.7 cases per 1 million individuals [4]. In the specific context of India, as of 2021, the reported rate was notably lower at 4 cases per million individuals, as documented by the WHO [13].

The recent large and disruptive measles outbreaks in 2022 were reported from 37 countries and the burden was largely concentrated in Africa, Eastern Mediterranean, European, and Southeast Asian regions [14]. The global decline in measles prevalence, along with related illnesses and fatalities, showcased positive trends for the last several decades. However, a notable shift in this pattern emerged between early 2018 and late 2019, marked by a threefold increase in measles cases during the first half of 2019 compared to the same period in 2018. In 2019, the reported surge in measles cases was the highest seen in the past two decades, resulting in a 50 % increase in measles-related deaths from 2016 to 2019, claiming 207,500 lives globally in 2019 alone [15]. This resurgence of measles outbreaks has been linked to underlying issues and gaps within routine immunization programs due to the COVID-19 pandemic [16], [17], [18], [19]. Given its highly contagious nature, measles vaccine coverage stands as a crucial measure reflecting gaps in healthcare systems, with lower coverage serving as a significant indicator of a heightened disease burden [19], [20].

India administers one of the world's largest immunization programs, targeting 26 million children and 30 million pregnant women [21]. Measles vaccination plays a crucial role in India's Universal Immunization Program (UIP) and was first introduced in 1985 for children aged 9–12 months. In 2010, a second dose of measles containing vaccine (MCV2) was introduced in the UIP, recommended for children aged 16–24 months. According to research findings, the two-dose measles vaccine demonstrates remarkable efficacy, and the occurrence of measles disease is uncommon among individuals who have received both doses, irrespective of their age [19]. According to the National Family Health Survey-5 (2019–2021), the coverage for MCV1 was 88.6 %, and for MCV2, it stood at 58.6 %. This represents substantial progress compared to NFHS-4 (2015–16) when MCV1 coverage was at 81.1 % [22]. However, the COVID-19 pandemic has posed challenges to vaccine coverage, including MCV1 and MCV2.

India’s Measles-Rubella Elimination Goal for 2020 was revised to 2023 due to the severe aftermath of the COVID-19 pandemic as a part of the South-East Asia Region (SEAR) elimination goal. WHO defines measles elimination as the absence of endemic measles in a defined geographical area for more than 12 months in the presence of a high-quality surveillance system followed by its sustenance for at least 36 months [23]. According to the recent recommendations from the India Expert Advisory Group on Measles and Rubella (IEAG-MR), each district needs to achieve at least 95 % MCV2 coverage for children by 2 or at least 5 years of age and achieve and maintain sensitive fever and rash surveillance and the elimination criteria must be followed across all the districts and regions in the country [24].

This paper explores the existing gaps in measles vaccination in India that would further inform the program components, decision-making, governance, stewardship, and program reach. After an extensive literature review, we found that research and publications at the global and national levels focused on the overall measles vaccination coverage limited to MCV1 in the context of full immunization coverage (FIC) up to one year of age. However, this paper aims to investigate dose-wise measles vaccination coverage – zero dose (no vaccination), one dose (partially vaccinated), and two doses (fully vaccinated) of measles vaccine among children aged 24–35 months mapped by sociodemographic characteristics utilizing the NFHS-5 data. NFHS-5 created a milestone by collecting data on MCV2 coverage in India, enabling an exploration of measles vaccination from a zero-dose perspective. The paper is aligned with the Immunization Agenda 2030 (IA 2030) with the objective that no one should be left behind and each child should be vaccinated with all recommended doses of vaccines. This has the potential to accurately enumerate the dose-wise critical gaps mapped against the sociodemographic variables and provide evidence for targeted action. The paper also aims to explore the spatial distribution of zero-dose measles vaccination and identify the states, Union Territories (UTs), districts, and clusters of regions with high zero dose burden to inform program recommendations. These insights are crucial for synthesizing information and prioritizing districts that can improve vaccination, reduce the measles burden, and achieve the elimination goal.

## 2 Data sources and methodology

### 2.1 Data source

For this study, we utilized data derived from the NFHS-5, 2019–21 internationally known as DHS data. NFHS-5 – a comprehensive nationwide survey conducted in households across India, that offers a wealth of information concerning fertility, infant and child mortality, maternal and child health, as well as various nutrition and health services, along with family welfare indicators, categorized by demographic traits, both at the national, state, and UT levels. NFHS-5 adopted a stratified two-stage sampling design. The urban and rural samples within each state were drawn separately. In each state, the rural sample was selected in two stages, with the selection of Primary Sampling Units (PSUs), which are villages, with probability proportional to population size (PPS) selection at the first stage, followed by random selection of households within each PSU in the second stage. In urban areas, a two-stage procedure was followed. In the first stage, census enumeration blocks (CEB) were randomly selected with PPS. In the second stage, households were randomly selected within each selected CEB. The survey was executed in two distinct phases – the first phase spanned from June 17, 2019, to January 30, 2020, encompassing 17 states and 5 UTs, while the second phase began on November 2020, to April 2021, covering 11 states and 3 UTs. In total, NFHS-5 collected data from 636,699 households, 724,115 women, and 101,839 men. For this study, 43,864 children aged 24–35 months in NFHS-5 were included. Under the UIP, a two-dose measles vaccine is recommended with an initial dose between 9 and 12 months of age, followed by the second dose between 16 and 24 months of age [22].

### 2.2 Outcome and exposure variables

The percentage distribution of measles vaccine coverage (zero dose, one dose, and two doses) among children aged 24–35 months by sociodemographic characteristics (exposure/predictor variables) is the outcome variable of interest. The exposure variables of interest were determined by reviewing the scientific systematic review literature on social determinants of childhood immunization in low and middle-income countries including birth order of children, age of children in months, wealth quintile, gender of the child, social group, religion, residence, mother’s education in years, place of delivery, media exposure, delivery by cesarean method and status of residing with husband [25]. The objective is to explore the relationship between measles vaccine coverage factored by the selected exposure variables and draw actionable recommendations that have the potential to inform programmatic interventions for the measles elimination goal.

### 2.3 Statistical analysis

The data analysis was performed using statistical software – Stata version 16.0 SE, Arc GIS 10.8, and Geoda. The weighted estimates of sociodemographic variables and outcome indicators were performed for the analysis. We implemented the survey design effect to reduce the error estimation due to sampling stages and the sampling method while estimating the percentages. This study analyzed the bivariate prevalence with Pearson’s chi-square test and p-values to assess the relationship between two categorical variables. Pearson’s chi-square test and p-values determine whether there is a significant association or dependence between the outcome variable and exposure variables. Multivariate multinomial logistic regression was used to analyze factors associated with childhood measles vaccines because of the hierarchical nature of the data set. The analysis uses the two-dose as a comparison group.

We employed multivariate multinomial logistic regression to examine the factors associated with childhood measles vaccine coverage, given the hierarchical structure of our dataset. We applied multilevel modeling to analyze data collected from various levels and the outcome was measured at the lowest level. We utilized multivariate multinomial logistic regression to assess associations across four levels: Level 1 (Individual), Level 2 (Primary Sampling Unit or PSU), Level 3 (District), and Level 4 (State), with a 95 % confidence interval (CI) and p-value. Before conducting the multivariate multinomial logistic regression, we checked for multicollinearity among predictors using a generalized variance inflation factor, which should ideally not exceed five. None of the predictors had a factor greater than four, indicating the absence of multicollinearity issues. The hierarchical nature of our survey data justified the use of multinomial modeling in this study.

$$\;\;logit\left(\frac{\pi_{\mathrm{icds}}^{\left(s\right)}}{\pi_{\mathrm{icds}}^{\left(t\right)}}\;\;\right)=\;\;\beta_{0}^{\left(s\right)}+\;\beta_{1}^{\left(s\right)}x_{1icds}+\cdots \;\;\;\beta_{n}^{\left(s\right)}x_{\mathrm{nicds}}\;+\alpha_{0cds}^{\left(s\right)}\;+v_{0ds}^{\left(s\right)}\;+u_{0s}^{\left(s\right)}$$

Here $\frac{\pi_{\mathrm{icds}}^{\left(s\right)}}{\pi_{\mathrm{icds}}^{\left(t\right)}}$ is the probability for the ith children categorical outcome variable i in the cluster c, district d, and state level s ($\pi_{\mathrm{icds}}^{\left(s\right)}$ = 1, 2 denotes failure or lack of occurrence of the event, while $\pi_{\mathrm{icds}}^{\left(t\right)}$ = 3 denotes success or the occurrence of the event). The parameter $\beta_{0}^{\left(s\right)}\;$ is the intercept (mean) of the absolute zero and one measle dose among children 24–35 months old and $\beta_{1}^{\left(s\right)}$ represents the effects of the explanatory variables on zero and one measles dose. The random intercepts regression model assumes that while the intercept or average outcome for individuals with a given set of characteristics varies between higher-level units, the relationship between the dependent and independent variables remains consistent across all contexts. Random-effects parameters $\alpha_{0cds}^{\left(s\right)}$ are the effect of the cluster, $v_{0ds}^{\left(s\right)}$ the effect of the district, and $u_{0s}^{\left(s\right)}$ the effect of state-level random effect or residuals error term. The residuals follow the assumption of independent and normally distributed errors with zero means and constant variances. The model estimates variances at different levels: $\alpha_{0cds}^{\left(s\right)}$ ∼ N (0,$\;\sigma_{c}^{2(s)}$) is within the district, between cluster variance; $v_{0ds}^{\left(s\right)}$ ∼ N (0, $\sigma_{c}^{2(s)}$) is within states, between-district variance and $u_{0s}^{\left(s\right)}$∼ N (0, $\sigma_{c}^{2(s)}$) represents between-state variance.

The overarching goal of multilevel models is to partition the variance in the outcome across hierarchical data levels. The Variance Partition Coefficient (VPC) is a statistical measure used to assess the proportion of variation in an outcome variable attributed to different factors or sources of variation. It is beneficial in hierarchical or mixed-effects models with multiple levels of nested data. The VPC is a straightforward measure, calculated as the ratio of the variance at a specific geographical level to the combined variance across all levels (1, 2 … N) [26]. This ratio quantifies the portion of variance in the outcome attributed to variations between hierarchical structures. The value of the variance of the underlying individual-level variable, according to the logistic distribution, is $\pi^{2}/3$ or 3.29.

$${\mathrm{VPC}}_{g}=\frac{\sigma_{g}^{2(s)}}{\left(\sigma_{s}^{2(s)}+\sigma_{d}^{2(s)}+\sigma_{c}^{2(s)}+\pi^{2(s)}/3\right)}$$

Where g represents a geographical area

### 2.4 Spatial analysis

In this study, we employed descriptive maps generated through ArcGIS, which were subsequently exported to GeoDa for spatial analysis. To facilitate our analysis, we employed a first-order contiguity matrix as a weight. Spatial autocorrelation was conducted to investigate whether the distribution of zero dose of measles vaccine exhibited a random pattern or not. Spatial autocorrelation, quantified by Moran's I, is akin to a Pearson correlation coefficient that assesses the relationship between a variable and its neighboring values within a geographic context. Moran's I is a spatial statistic used to gauge the presence of spatial autocorrelation in an entire dataset, yielding a single output value. The Moran's I value ranges from −1 to 1, with negative values indicating negative spatial autocorrelation and positive values indicating positive spatial autocorrelation. Positive autocorrelation suggests that areas with similar characteristics are clustered closely in space, whereas negative spatial autocorrelation suggests that closely located areas exhibit dissimilar characteristics. A value of Moran's I close to 0 implies the absence of spatial autocorrelation [27].

## 3 Results

### 3.1 Scenario of measles vaccination in India

The findings in the Table 1 provides valuable insights into the burden of zero-doses of measles vaccination among children in the study population. The data revealed a higher burden of zero-doses among children with 4th or higher birth orders, accounting for 17.2 % of the cases. Children from the poorer wealth quintile exhibited a higher prevalence of 15.9 % zero-dose. Conversely, children in the richest wealth quintile have a lower burden, at only 8.8 %. No significant gender or social differences in the burden of zero-dose were observed. The data indicated that children from the Muslim community experienced a higher burden of zero-doses, with a prevalence of 16.6 %. Interestingly, no significant differences in the burden of zero-doses were found between rural and urban areas. Maternal education emerged as a significant determinant, with children of mothers having 12 or more years of education reflecting a lower burden of zero-doses (8.9 %). In contrast, children of mothers with no education exhibited a higher burden at 17.8 %. A notable determinant that emerged was maternal media exposure where children of mothers with no media exposure displayed a higher burden of zero-doses (14.4 %) while children of mothers with media exposure had a lower burden (8.8 %). Overall, 11.5 % of children are measles zero-dose in India, with 29.8 % partially vaccinated and 58.6 % having completed their two doses of recommended vaccination against measles.

**Table 1:** Percentage distribution of measles vaccine coverage among children aged 24–35 months by sociodemographic characteristics in India, NFHS-5 (2019–21).

**Independent Variables**	**Zero-Dose**		**One-Dose**		**Two-Dose**		CHi2	P value	Sample
**Birth Order**	%	[95 % CI]	%	[95 % CI]	%	[95 % CI]			
1st	9.4	[8.7,10.2]	29.0	[28.0,30.0]	61.6	[60.5,62.7]	1490.399	<0.001	17,005
2nd −3rd	11.8	[11.1,12.5]	30.4	[29.6,31.3]	57.8	[56.8,58.8]			21,626
4th and more	17.2	[15.9,18.6]	30.4	[28.7,32.0]	52.4	[50.7,54.2]			5234

**Children age in months**									
24–27	11.0	[10.3,11.8]	31.3	[30.3,32.3]	57.7	[56.6,58.8]	131.0065	0.003	15,187
28–35	11.8	[11.1,12.5]	29.1	[28.3,29.8]	59.1	[58.3,60.0]			28,677

**Wealth index**									
Richest	8.8	[7.5,10.4]	28.4	[26.8,30.1]	62.8	[60.9,64.6]	1868.018	<0.001	7236
Rich	9.3	[8.2,10.6]	29.6	[28.1,31.2]	61.1	[59.4,62.7]			8244
Middle	10.2	[9.3,11.2]	30.0	[28.7,31.3]	59.8	[58.3,61.2]			8351
Poorer	11.8	[11.0,12.7]	30.3	[29.1,31.5]	57.9	[56.6,59.2]			9449
Poorest	15.9	[14.9,16.8]	30.5	[29.4,31.6]	53.6	[52.3,54.9]			10,584
Gender of child									
Male	11.7	[11.0,12.3]	29.3	[28.5,30.1]	59.1	[58.1,60.0]	37.0911	0.151	22,742
Female	11.4	[10.7,12.1]	30.4	[29.6,31.3]	58.2	[57.2,59.1]			21,123

**Social group**									
Others	12.2	[10.7,13.9]	30.6	[29.2,32.0]	57.2	[55.5,59.0]	282.7757	<0.003	10,294
Other Backward Caste	11.1	[10.5,11.8]	29.6	[28.7,30.5]	59.3	[58.3,60.3]			19,032
Schedule tribe	12.4	[11.2,13.6]	32.8	[31.1,34.5]	54.8	[53.0,56.7]			4267
Schedule caste	11.2	[10.4,12.2]	28.4	[27.1,29.6]	60.4	[59.0,61.8]			10,272

**Religion**									
Hindu	10.5	[9.9,11.1]	29.3	[28.6,30.0]	60.2	[59.4,61.0]	1616.651	<0.001	34,779
Muslim	16.6	[15.4,18.0]	31.1	[29.5,32.7]	52.3	[50.4,54.1]			7163
Christian	13.4	[11.4,15.7]	37.3	[33.5,41.2]	49.3	[45.2,53.5]			918
Others	10.4	[8.0,13.3]	32.5	[28.6,36.6]	57.2	[53.1,61.1]			1005
**Residence**									
Urban	11.1	[10.1,12.1]	29.0	[27.6,30.5]	59.9	[58.3,61.4]	56.662	0.161	11,951
Rural	11.7	[11.1,12.3]	30.1	[29.5,30.8]	58.2	[57.4,59.0]			31,913

**Mother education in years**									
12 or more years	8.9	[7.0,11.2]	27.7	[26.0,29.4]	63.5	[61.3,65.6]	3154.447	<0.001	7208
9–12 year	8.8	[8.2,9.5]	29.9	[28.9,31.0]	61.2	[60.1,62.4]			14,755
5–8 years	11.3	[10.5,12.1]	30.6	[29.4,31.8]	58.1	[56.8,59.4]			10,582
<5 years	12.9	[11.1,15.1]	30.8	[28.2,33.4]	56.3	[53.4,59.2]			2114
No schooling	17.8	[16.8,18.9]	30.3	[29.1,31.6]	51.8	[50.5,53.2]			9206

**Place of delivery**									
Private health facility	10.7	[9.3,12.1]	30.2	[28.9,31.6]	59.1	[57.5,60.7]	2582.216	<0.001	11,918
Public health facility	10.3	[9.8,10.8]	29.5	[28.7,30.2]	60.2	[59.3,61.0]			27,317
No health facility	20.8	[19.3,22.3]	31.0	[29.3,32.7]	48.2	[46.3,50.1]			4630

**Media exposure at least once a week**									
No	14.4	[13.7,15.0]	30.9	[30.1,31.7]	54.7	[53.8,55.7]	2244.653	<0.001	21,576
Yes	8.8	[8.0,9.6]	28.8	[27.9,29.7]	62.4	[61.4,63.4]			22,289

**Delivery by caesarean**									
No	12.4	[11.8,13.0]	30.3	[29.6,31.0]	57.3	[56.5,58.1]	894.7197	<0.001	34,257
Yes	8.3	[7.5,9.3]	28.2	[26.8,29.6]	63.5	[62.0,65.0]			9608

**Currently residing with husband**									
Living with her	11.2	[10.7,11.8]	29.7	[29.1,30.4]	59.0	[58.3,59.8]	167.0965	<0.001	37,853
Staying elsewhere	13.5	[12.2,14.8]	30.5	[28.9,32.1]	56.1	[54.3,57.9]			6011
**Total**	**11.5**	**[11.0,12.1]**	**29.8**	**[29.2,30.5]**	**58.6**	**[57.9,59.4]**			**43,864**

### 3.2 Geographical distribution and spatial clustering of measles zero-dose

**Fig. 1:**
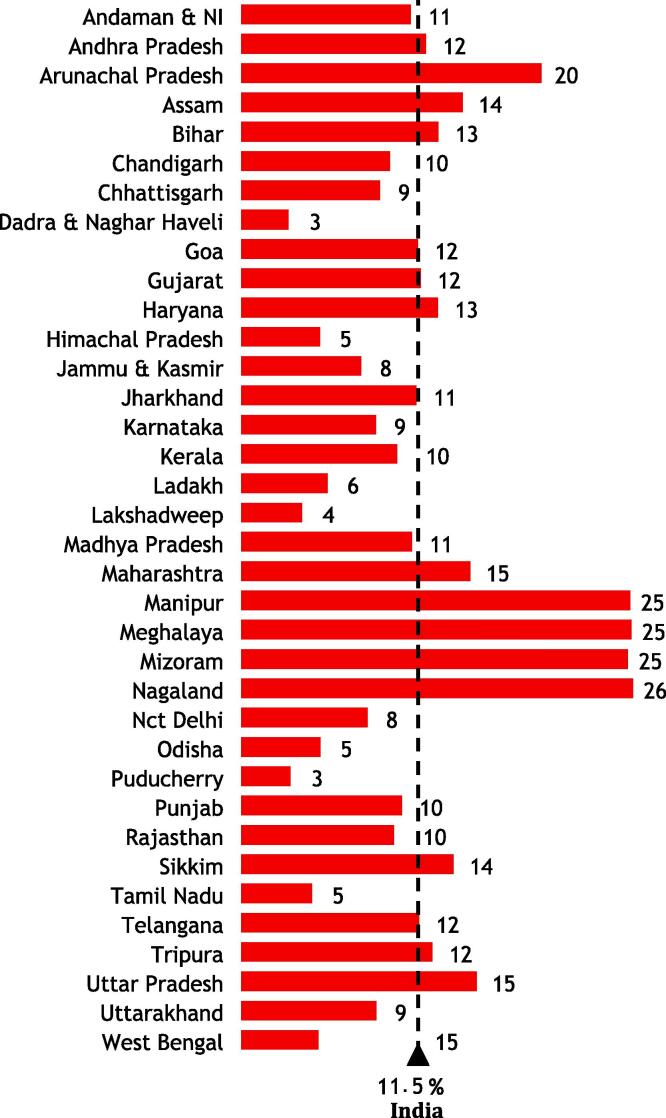
Prevalence of zero-dose of measles vaccine coverage by states in India.

**Fig. 2:**
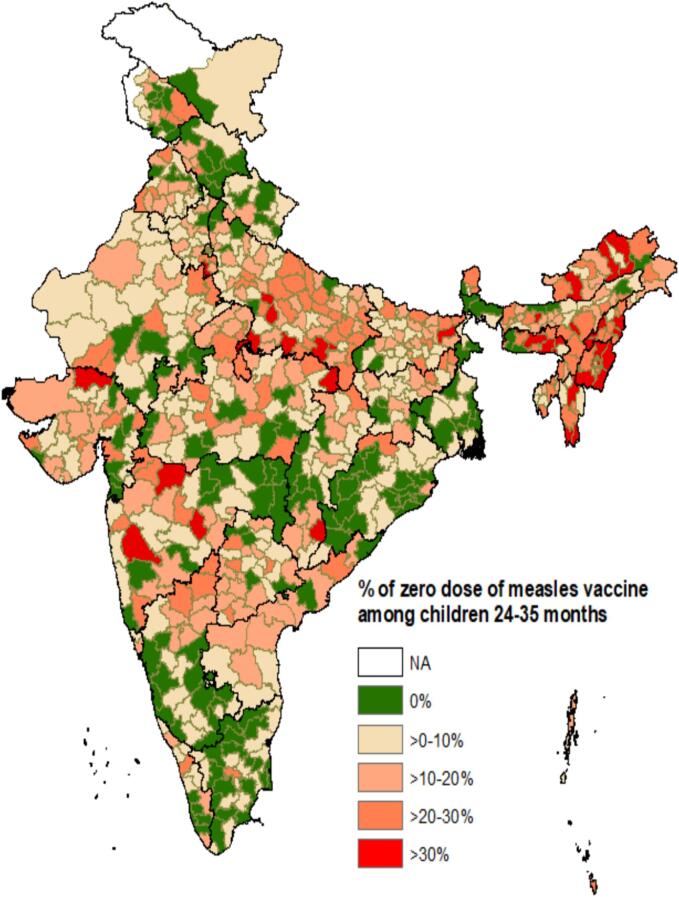
Prevalence of zero-dose of measles vaccine coverage by districts in India.

**Fig. 3:**
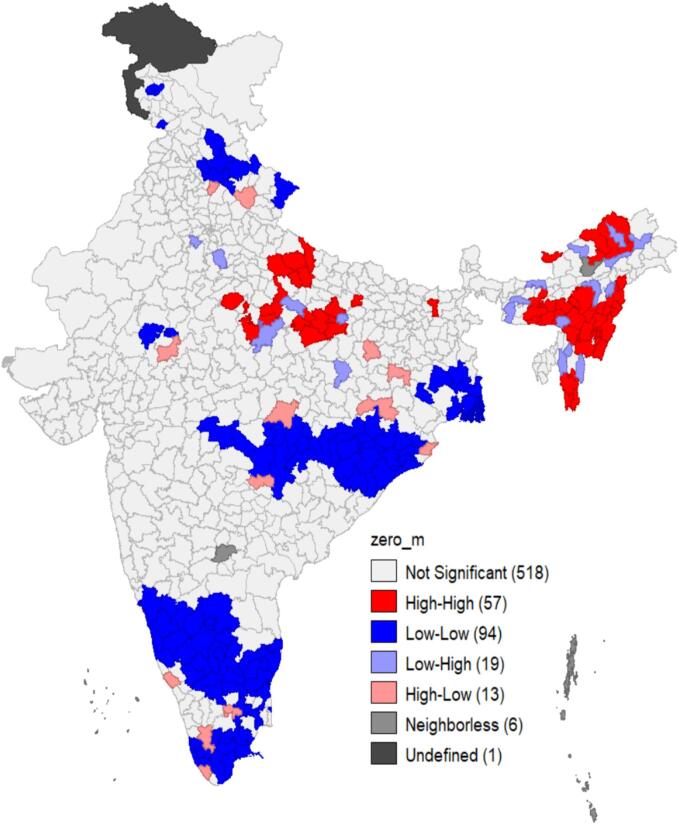
Univariate LISA maps clustering of zero-dose measles.

The Fig. 1 depicts the prevalence of zero-dose measles vaccine by states in India. While the country’s zero-dose prevalence stands at 11.5 %, 16 states still have higher zero-dose prevalence than the national data. All 8 northeastern states in India have shown higher measles zero dose than the national measles zero dose data. Out of these 8 states, 5 states have measles zero-dose either 20 % or more with Nagaland at 26 %. Fig. 2 further delves down into the prevalence of measles zero-dose of vaccine mapped district-wise across the nation. In total, 26 districts have more than 30 % measles zero-dose children. Primarily the districts in the north-eastern region (constitutes 8 states – Assam, Arunachal Pradesh, Manipur, Meghalaya, Mizoram, Nagaland, Sikkim, and Tripura), a few districts in Maharashtra and Uttar Pradesh, and 1–2 districts in Madhya Pradesh, Chhattisgarh, Bihar, Gujarat, and Haryana have more than 30 % measles zero dose children. Fig. 3 illustrates the LISA cluster map of measles zero-dose that are primarily present in northeastern regions, Uttar Pradesh, and Madhya Pradesh – indicated in red regions that are significant positive. This correlates with the interpretation drawn from the Fig. 1, Fig. 2. The map also portrays clusters of regions with low measles zero-dose prevalence – indicated in blue regions that are also significant positive. The paler regions (pale blue and pink) are less significant and the grey regions are insignificant.

### 3.3 Risk factors and probability of measles zero-dose

Among children aged 24–35 months, the Table 2 shows those who were fourth or later in birth order had 1.21 times higher odds of not receiving any doses of the measles vaccine compared to children who were first in birth order (OR = 1.12, 95 % CI: 1.05, 1.20). Children aged 28–35 months were 0.92 times less odds to receive one dose of the measles vaccine than children aged less than 28 months (OR = 0.92, 95 % CI: 0.88, 0.96). Children from the poorest families had 1.66 times higher odds of not receiving any doses of the measles vaccine (OR = 1.66, 95 % CI: 1.43, 1.94) and 1.21 times higher odds of receiving only one dose of the measles vaccine (OR = 1.21, 95 % CI: 1.09, 1.34) compared to children from the wealthiest families. Among children classified as Other Backward Class (OBC), there was 0.89 times lower odds of not receiving any doses of the measles vaccine (OR = 0.89, 95 % CI: 0.81, 0.97) and 0.92 times lower odds of receiving only one dose of the measles vaccine (OR = 0.92, 95 % CI: 0.86, 0.98) compared to children from other social groups. Muslim children were 1.54 times the odds not to receive any doses of the measles vaccine (OR = 1.54, 95 % CI: 1.40, 1.70) and 1.09 times the odds to receive only one dose of the measles vaccine (OR = 1.09, 95 % CI: 1.01, 1.17) compared to children from the Hindu religion. Children residing in rural areas were 0.78 times less odds to not receive any doses of the measles vaccine compared to urban children (OR = 0.78, 95 % CI: 0.71, 0.86). Among children whose mothers had no education, there was 1.54 times higher odds of not receiving any doses of the vaccine (OR = 1.54, 95 % CI: 1.35, 1.76) and a 1.14 times higher odds of receiving only one dose of the measles vaccine (OR = 1.14, 95 % CI: 1.05, 1.25) compared to children whose mothers had more than 12 years of education. Children who were not delivered at a health facility (either public or private) had 1.62 times higher odds of not receiving any doses of the measles vaccine and 1.10 times higher odds of receiving only one dose of the measles vaccine compared to children who were delivered in private health facilities. Among children whose mothers had media exposure (such as reading newspapers, listening to the radio, or watching television) at least once a week, there was a 0.79 times lower odds of not receiving any doses of the measles vaccine (OR = 0.79, 95 % CI: 0.73, 0.84) and a 0.94 times lower odds of receiving only one dose of the measles vaccine (OR = 0.94, 95 % CI: 0.90, 0.99) compared to children whose mothers had no media exposure. Children delivered by C-section had 0.90 times lower odds of not receiving any doses of the measles vaccine (OR = 0.90, 95 % CI: 0.82, 0.99) and 0.92 times lower odds of receiving only one dose of the measles vaccine (OR = 0.92, 95 % CI: 0.87, 0.98) compared to children who were born through normal delivery. Among children whose fathers did not reside with their families, there was a 1.11 times higher odds of not receiving any doses of the measles vaccine (OR = 1.11, 95 % CI: 1.01, 1.21) and a 1.09 times higher odds of receiving only one dose of the measles vaccine (OR = 1.09, 95 % CI: 1.02, 1.16) compared to children whose fathers resided with them.

**Table 2:** Multilevel multivariate multinomial logistic regression of measles vaccine coverage among children aged 24–35 months with sociodemographic characteristics in India, NFHS-5 (2019–21).

**Independent Variables**	**Zero- Dose**		**One-Dose**	
**Birth Order**	OR	[95 % CI]	OR	[95 % CI]
1st				
2nd −3rd	1.12***	[1.05,1.20]	1.07**	[1.03,1.12]
4th and more	1.21***	[1.10,1.33]	1.02	[0.95,1.10]

**Children age in months**				
24–27				
28–35	1.01	[0.95,1.08]	0.92***	[0.88,0.96]

**Wealth index**				
Richest				
Rich	1.15*	[1.01,1.32]	1.03	[0.95,1.12]
Middle	1.32***	[1.15,1.51]	1.12**	[1.03,1.22]
Poorer	1.41***	[1.22,1.63]	1.14**	[1.04,1.25]
Poorest	1.66***	[1.43,1.94]	1.21***	[1.09,1.34]

**Gender of child**				
Male				
Female	0.96	[0.91,1.02]	0.99	[0.95,1.03]

**Social group**				
Others				
Other Backward class	0.89*	[0.81,0.97]	0.92**	[0.86,0.98]
Schedule tribe	1.01	[0.89,1.14]	1.01	[0.92,1.10]
Schedule caste	0.92	[0.83,1.03]	0.93*	[0.86,1.00]

**Religion**				
Hindu				
Muslim	1.54***	[1.40,1.70]	1.09*	[1.01,1.17]
Christian	1.35***	[1.13,1.61]	1.05	[0.92,1.20]
Others	1.1	[0.90,1.33]	0.97	[0.84,1.11]

**Residence**				
Urban				
Rural	0.78***	[0.71,0.86]	0.98	[0.92,1.04]

**Mother education in years**				
12 or more years				
9–12 year	0.97	[0.86,1.09]	1.02	[0.95,1.09]
5–8 years	1.13	[1.00,1.29]	1.08	[0.99,1.16]
<5 years	1.27**	[1.08,1.50]	1.07	[0.95,1.20]
No schooling	1.54***	[1.35,1.76]	1.14**	[1.05,1.25]

**Place of delivery**				
Private health facility				
Public health facility	0.88**	[0.80,0.96]	0.98	[0.93,1.05]
No health facility	1.62***	[1.44,1.82]	1.10*	[1.01,1.20]

**Media exposure at least once a week**				
No				
Yes	0.79***	[0.73,0.84]	0.94*	[0.90,0.99]

**Delivery by caesarean**				
No				
Yes	0.90*	[0.82,0.99]	0.92**	[0.87,0.98]

**Currently residing with husband**				
Living with him				
Staying elsewhere	1.11*	[1.01,1.21]	1.09*	[1.02,1.16]

Note- Significance: *P < 0.05; **P < 0.01; ***P < 0.001.

Table 3 shows the multilevel distribution of the Variance Partition Coefficients (VPCs) of the zero-dose and one dose of measles vaccine among children aged 24–35 months in India. Outcomes reflected that 3.08 % of the variation in zero-dose measles vaccine lies between states, 5.02 % lies within states between districts, and 9.40 % lies within districts between clusters. This analysis also estimated the VPC of one dose of measles vaccine, 7.45 % of the variation in one dose of measles vaccine lies between states, 2.27 % lies within states between districts, and 5.29 % lies within districts between clusters. Table 4 shows the Moran's I value is 0.33 and is positive and it shows that the regions with high values are surrounded by regions with high values.

**Table 3:** Multilevel multinomial distribution of the Variance Partition Coefficients (VPCs) of zero dose and one dose of measles vaccine coverage among children aged 24–35 months in India, NFHS-5 (2019–21).

	Measles vaccine	
Geographies	Zero-dose	One dose
	VPC in %, (95 % C.I)	VPC in %, (95 % C.I)
States	3.08, (1.31, 4.68)	7.45, (3.92, 10.61)
District	5.02, (4.19, 5.76)	2.27, (1.89, 2.60)
Cluster	9.40, (7.65, 10.99)	5.29, (4.40, 6.09)

**Table 4:** Moran's I for the zero dose of measles vaccination.

**Selected Variables**	**Moran's I**
Univariate	
Zero dose measles	0.339935

## 4 Discussion

Measles vaccination continues to be a significant public health concern in India due to the substantial burden of measles zero-dose children. This paper investigated critical aspects of measles vaccination that were previously overlooked, specifically focusing on zero-doses, partially vaccinated, and those who were fully immunized. The results reveal noteworthy insights – 11.5 % of eligible children in India have not received any dose of measles vaccination, while 29.8 % have received only MCV1, and 58.6 % are vaccinated with both MCV1 and MCV2 doses. Furthermore, the findings highlight considerable variations between states and districts regarding zero-dose (measles) prevalence. Nagaland exhibits the highest distribution of zero-dose cases at 26 % while Tamil Nadu reports the lowest at 4.6 %. A closer look at the district-level results within a state reveals that West Siang district in Arunachal Pradesh has the highest percentage with 49.6 % of children classified as zero-dose cases; however, Lower Dibang Valley district has only 2.8 % percent of zero-dose children. Another major cluster region that emerged was in Uttar Pradesh with huge inter-district variations – Prayagraj and Banda districts have 34.2 % and 32.2 % while Hapur and Etawah have 2.6 % and 2.1 % zero dose children. These findings call for evidence-based and context-specific interventions for respective states, districts, and clusters of regions.

The measles vaccination status of children was significantly associated with key sociodemographic factors – birth order, wealth quintile, gender, mother’s education, media exposure, place of delivery, social group, religion, etc. Notably, children with higher birth orders and those from the poorest wealth quintile exhibited a higher percentage of zero-doses. Mothers with lower levels of education showed an increased odd of having zero-dose measles children. Additionally, mothers with limited media exposure demonstrated a higher probability of their children having a zero-dose status for measles. There can be several potential reasons but the main reason could be parents might be less vigilant about following immunization schedules for subsequent children compared to their firstborn. They might assume they already know the process or underestimate the importance of vaccination. The children from lower wealth quintiles have the lowest immunization coverage which may be due to a combination of factors including awareness, and challenges balancing time and resources for multiple vaccinations. Increased media exposure might give information and awareness on the public health benefits of vaccination (and vice versa) and that this association with limited media exposure and high OR for having a zero-dose measles vaccination status. The vaccine hesitancy among some Muslim and Christian communities, may be influenced by cultural factors and misinformation leading to lower vaccination rates. These findings emphasize the intricate interplay between socio-demographic factors and measles vaccination status and how these interpretations could potentially inform interventions and hence should be considered while planning. These findings are aligned with other similar works on the measles vaccination nationally and globally [28], [29], [30], [31]. The Variance Partition Coefficients reflected variations between states, districts, and clusters with intra-district dynamics at the cluster level emerging as the most prominent – 9.4 % of the variation in zero-dose measles vaccine. This underscores the critical role of contextual interventions within districts, emphasizing the need for tailored strategies to address specific clusters and communities. Conversely, the prominence of state-level factors in explaining 7.45 % of the variation for one dose highlights the need for broader statewide initiatives. The clusters of measles zero-dose are largely concentrated in the northeastern, northern (Uttar Pradesh), and central (Madhya Pradesh) regions. The persistently lower vaccination coverage of routine immunization in these regions coupled with higher fertility rates presents a unique challenge [32], [33]. The measles transmission is higher in these regions and intensifies the struggle to maintain high routine immunization coverage. Studies have identified that these regions and districts show lower routine immunization performance and coverage and high zero-dose prevalence [34]. The findings of this paper substantiate the importance of spatial data in guiding immunization programs and disease elimination.

Spatial justice and equity are fundamental principles emphasizing the importance of equitable access to healthcare resources for all individuals regardless of zip code or socioeconomic status [35]. Disparities in vaccination coverage hinder progress toward disease control and elimination. Spatial analysis allows for the identification of clusters of unvaccinated and under-vaccinated populations guiding resource allocation for targeted interventions [36]. WHO’s IA 2030, a global health policy framework recognizes the importance of spatially detailed data in achieving high and equitable vaccination coverage [37]. The Global Measles and Rubella Strategic Framework 2021–30 (MRSF 2021–30) – a framework to guide stakeholders at regional and national levels to achieve and sustain measles elimination is in alignment with the IA 2030, UNICEF Immunization Roadmap 2018–30, and Gavi Alliance 2021–25 Strategy (Gavi 5.0) and focuses on defining the priorities and making essential program pivots [35]. MRSF 2021–30 also recommends shifting to tailored approaches instead of non-selective mass campaigns with an aim to bring zero-dose and partially vaccinated children within the ambit of health systems possibly left out due to the COVID-19 pandemic disruptions. Targets 3.2 and 3.8 under Sustainable Development Goal (SDG) 3 for health state that countries are aiming to reduce the neo-natal mortality rates and working towards the provision of access to safe, effective, quality, and affordable vaccines for all respectively, and the measles elimination goal is linked to both targets [38].

In 2017, India adopted the ‘National Strategic Plan for Achieving and Sustaining Measles and Rubella Elimination’, and since then the country has vaccinated more than 348 million children through the measles rubella (MR) vaccination campaigns [13], [39]. In addition, these catch-up campaigns were designed to vaccinate all children from 9 months up to 15 years of age across targeted districts irrespective of the MCV vaccination status and previous history of illness. The target of reducing the number of measles zero-dose children significantly by 2030 aligns with the findings from various studies, emphasizing the need to reach at least 95 % coverage for both doses of measles vaccine. The unvaccinated (measles zero-dose) children pose an immediate health risk, amplify disease transmission, and act as a barrier to the measles elimination goal. In September 2022, India adopted a ‘Roadmap to Measles and Rubella Elimination in India’ which serves as a guidance document for all 36 states/UTs and includes measurable goals and timelines, prioritization, accountability, and sustainability mechanisms [17], [40].

Measles outbreak is considered an early warning sign for immunization programs and can be effectively used as a signal for tracing missed and dropout children and overall systems strengthening [41]. It is an ideal tracer as measles outbreaks visibly signal clusters with suboptimal immunization service delivery and can drive prioritization of targeted interventions to improve program performance and advocacy [42]. The COVID-19 disruptions also uncovered the systemic gaps and the vulnerable populations were at a heightened risk of measles and other vaccine-preventable disease [43]. India responded resiliently to the pandemic through the phased resumption of immunization services and optimization of UIP’s framework. However, the measles zero-dose burden remains a critical gap in the elimination goal. This is primarily referred to as a ‘diagonal approach’ wherein measles disease transmission indicates susceptible regions and possible areas for systemic improvements including other vaccine-preventable diseases and health goals [44].

Recent measles outbreaks in India were reported in 2022 from several districts of Maharashtra, Bihar, Gujrat, Haryana, Jharkhand, Kerala, and Delhi and measles-related deaths were also recorded subsequently [40], [45], [46], [47]. The Government of India's (GoI) response to the outbreak was based on the 4th IEAG-MR recommendations from May 2022, and the government launched an ‘Outbreak Response Immunization’ (ORI) drive. ORI focused on ramping up immunization sessions, conducting intensive community awareness initiatives, intensifying detection, and surveillance, and ensuring clinical management of cases. Active fever and rash surveillance were recommended for early detection of measles cases particularly in the regions that experienced an outbreak. A series of national and state-level workshops were conducted to improve the capacity of government and FLWs to manage outbreaks and conduct immunization drives. Spatial clustering of unvaccinated individuals and densely populated areas increases the risk of such outbreaks and these recent incidences exemplify the need for spatial evaluation of measles disease burden [43]. Socioeconomic factors play a significant role in measles transmission, with individuals from lower socioeconomic classes experiencing higher attack rates resulting in outbreaks [28], [48].

Lessons from past pandemics including COVID-19, the successful global eradication of Smallpox, and the Polio eradication journey in most countries have similarities and shared goals in common [49]. All these were aimed at achieving and maintaining high-population immunity and simultaneously prioritizing the regions with high disease burden and low vaccination coverage. The strategies for measles and rubella elimination share similarities with polio eradication – high levels of population immunity (∼95 %) with both doses of MCV, monitoring disease using sustained and effective surveillance and laboratory network, implementing outbreak preparedness plan, prioritizing public health communications, building vaccine confidence and demand for vaccination, need-based supplementary immunization activities (SIA) for high-risk areas and conflicting populations and conducting research for program improvement [20], [50], [51]. The assets and lessons from polio can help shape measles elimination strategies and further inform program recommendations. Humans being the only reservoir for the measles virus and no documented evidence of asymptomatic carriers makes its elimination even more possible [19]. The polio eradication framework and certification mechanism could also guide the measles elimination certification processes including metrics for evaluation [49].

Integration of measles vaccination within broader child health programs is crucial. Given the specific timelines for the first and second doses of measles vaccine, typically administered between 9 to 12 months and 16 to 24 months respectively; aligning measles vaccination with early childhood care initiatives becomes paramount. The initial months often witness heightened parental attention to vaccinations and overall child well-being, gradually tapering off as the child grows older. Therefore, integrating measles vaccination with comprehensive child health programs can ensure sustained awareness and coverage, providing a holistic approach to safeguarding children against preventable diseases beyond the infancy stage.

The country could possibly move towards exploring the utilization of microarray patches as a long-term approach. The immune response triggered by the vaccine irrespective of the mode of administration – sub-cutaneous or patches remains the same [52]. Measles and Rubella Microarray Patches (MR-MAPs) have the potential to contribute to the path towards elimination through easy door-to-door administration. The current Needle and Syringe (N&S) method requires a skilled healthcare worker and has opportunities for logistical failures related to its storage, transportation, reconstitution, and administration. Some of the potential programmatic benefits are – relatively painless, more acceptable, can be administered by healthcare workers with limited training, has thermostability with the potential to be administered in remote settings, and does not require reconstitution [53]. All this together can overcome logistical challenges leading to zero-dose children and inequitable MR coverage. However, it is important to understand the need for MR-MAPs in challenging settings in India and in what ways the existing health system could be used to ensure its procurement and implementation.

Measles cases is increasingly concentrated in areas with the lowest vaccination coverage. MCV2 introduction creates new opportunities to deliver other child health interventions beyond the first year of life, and it can provide an opportunity to catch up on missed vaccines and improve coverage with all recommended vaccines. MCV2 coverage should be used as a key performance indicator for EPI. School entry vaccination checks and laws supporting vaccination requirements have proven to be a highly effective strategy for increasing vaccination coverage and preventing outbreaks and were critical to strategies that achieved measles elimination in the United States [51]. Despite the challenges posed by the COVID-19 pandemic India has made remarkable progress in the elimination of measles and rubella. Improved MR surveillance after the second COVID-19 wave is a testament to India's dedication towards elimination. Addressing zero-dose children and improving measles vaccination coverage is pivotal for achieving the measles elimination goal and safeguarding the health of vulnerable populations. Any disease eradication or elimination effort begins and ends with the need for high-quality data and surveillance mechanisms. With consistent efforts, the country aims to catch up on the immunization gaps and vaccinate dropped-out and left-out children this year through Intensified Mission Indradhanush (IMI) 5.0 campaigns. So far 6 phases of Intensified Mission Indradhanush (IMI) have been conducted from 2017 to 2022 with a focus on measles rubella (MR) elimination vaccinating approximately 1.9 million children [54]. This year, IMI 5.0 commenced in August 2023 and it aimed to vaccinate all children up to the age of 5 years as compared to previous cohorts of up to 2 years across the country. The government also introduced U-WIN – a name-based immunization registry system serving as a single source of truth for monitoring immunization across all states and UTs [55]. This elimination approach including spatially targeted interventions and strengthened health systems is essential to overcome these challenges.

## 5 Limitations

Robust vaccination coverage data encompassing all eligible children and the identification of susceptible subpopulations are essential for crafting targeted immunization strategies. Relying solely on coverage survey data, even at the subnational level, may prove insufficient in pinpointing subpopulations characterized by low immunization rates and heightened susceptibility. The study encounters limitations in establishing a correlation between measles vaccination coverage and recent measles outbreaks due to a lack of district-wise measles outbreak data. The multifaceted reasons behind under-vaccination pose a challenge, and qualitative interviews emerge as a valuable avenue for gaining deeper insights into this complex issue of zero-dose children for measles vaccination. The study also acknowledges a limitation in the data due to recall bias. According to NFHS-5 data, among mothers of children aged 24–35 months, 82.68 % have an immunization card, which is verified by the enumerator. Additionally, 10.72 % of mothers have a card but are unable to produce it, while 6.65 % of mothers do not possess any vaccination card. This indicates that 17 % of the immunization related information relies on mothers' recall potentially impacting the quality of the data.

## 6 Conclusion

This study sheds light on critical facets of measles vaccination in the context of the measles elimination goal. The identification of a notable percentage (11.5 %) of children receiving zero-doses signals a concerning gap in immunization coverage. This highlights the importance of last mile effort and application of big push theory in terms of more frequent rounds of campaigns mode vaccine delivery for target of 95 % vaccine coverage for population level immunity. The multilevel analysis unraveled distinct patterns, emphasizing the significance of localized interventions. Our findings underscore the need for tailored strategies, particularly at the cluster (Primary health center and subcenter level) within districts, where 9.40 % of the variability in zero-dose measles vaccines is observed. Understanding and addressing this variability could be pivotal in closing the immunization gap and preventing measles outbreaks in specific areas. Furthermore, the association between vaccination status and socio-demographic factors such as birth order, wealth quintile, maternal education, media exposure, religion, and maternal education reveals nuanced challenges. Policymakers and health practitioners should consider these socio-economic and cultural contexts while designing interventions hence influencing vaccination decisions.

## Author contributions

Conceived and designed the research paper: P.D, P.K, A.V, and A.K.S; analyzed the data: A.V and A.K.S; Wrote the manuscript: A.K.S and P.P; Refined the manuscript: G.T, B.T and P.P.

## Funding

This work was supported by the Bill & Melinda Gates Foundation through grant ID: INV-005893.

## CRediT authorship contribution statement

**Pritu Dhalaria:** Conceptualization. **Pawan Kumar:** Conceptualization. **Ajay Verma:** Formal analysis, Conceptualization. **Pretty Priyadarshini:** Writing – review & editing, Writing – original draft. **Ajeet Kumar Singh:** Writing – review & editing, Writing – original draft, Formal analysis, Conceptualization. **Bhupendra Tripathi:** Supervision. **Gunjan Taneja:** Writing – review & editing, Supervision.

## Declaration of competing interest

The authors declare the following financial interests/personal relationships which may be considered as potential competing interests: Pritu Dhalaria reports financial support was provided by Bill & Melinda Gates Foundation. If there are other authors, they declare that they have no known competing financial interests or personal relationships that could have appeared to influence the work reported in this paper.

## Data Availability

Data will be made available on request.
